# Editorial: Artificial intelligence and advanced technologies in neurological surgery

**DOI:** 10.3389/fsurg.2023.1251086

**Published:** 2023-07-18

**Authors:** Jessica Ryvlin, John H. Shin, Reza Yassari, Rafael De la Garza Ramos

**Affiliations:** ^1^ Department of Neurological Surgery, Montefiore Medical Center, Albert Einstein College of Medicine, Bronx, NY, United States; ^2^ Department of Neurological Surgery, Massachusetts General Hospital, Harvard Medical School, Boston, MA, United States

**Keywords:** neurosurgery, machine learning and artificial intelligence, patient outcomes, technology, imaging, minimally invasive & robotic surgery

**Editorial on the Research Topic** Artificial intelligence and advanced technologies in neurological surgery

## Introduction

Surgical treatment of neurologic disorders has always benefited from technologic advancements in operative techniques and equipment, imaging, predictive analytics, and many others. In recent years, almost all neurosurgical subspecialties have seen a substantial effort to develop, validate, and utilize artificial intelligence and machine learning models in a clinical manner (1). Unique patient cohorts including those undergoing intervention for primary brain tumors (2), spinal deformity and metastatic disease (3, 4), and trauma (5), have been studied extensively to generate tools and models with the main goal of optimizing patient outcomes. Patient-specific risk factors have additionally been identified using these algorithms, which are now being incorporated into accessible online calculators for prognostication and operative planning (6). Apart from artificial intelligence, improvements in imaging capability and incorporation of robotics has led to the development of new operative techniques designed to mitigate poor outcomes and improve postoperative recovery. Minimally invasive surgery, intraoperative navigation, and 3D reconstructions are now becoming frequently utilized in hospitals around the world, promising greater anatomical accuracy, decreased radiation exposure, and shorter operative times (7). As neurosurgical technology rapidly advances, we will similarly see a greater shift towards high quality personalized care, potentially at significant economic costs and steep learning curves initially.

In this research topic, we explore recent technologic advancements made in neurosurgery, including supervised machine learning models aimed at predicting postoperative survival in glioma patients, customized 3D printing and laser navigation applied to minimally invasive hematoma evacuation, an advanced cerebrospinal fluid (CSF) drainage method for use in posterior fossa tumor resection, and a novel endoscopic approach for treatment of oculomotor nerve palsy, all of which may help to inspire future work in similar topics of interest.

## Review of selected articles

At the forefront of advancements in neurosurgical outcomes analysis is artificial intelligence and machine learning. A retrospective cohort study conducted by Li et al. at an academic institution in China compared the accuracy of traditional statistical models and supervised machine learning models in predicting patient survival following resection for gliomas. Multivariate Cox proportional hazards regression, support vector machine (SVM), random survival forest (RSF), and tree and component gradient boosting (GB) models were evaluated using patient-specific clinical and biomarker variables and were subsequently compared in performance using concordance indexes. Predictably, the traditional Cox proportional hazards regression model performed poorly in both training and testing sets when compared to the supervised machine learning models, of which the GB models produced superior performance in predicting survival. Time-dependent survival performance was additionally assessed between tree GB and component GB models, which displayed good discrimination (area under the curve >0.8) at all evaluated time points. The authors of this study recognized many important patient variables found to influence survival based on these models, including functional status, tumor size, and resection type, which can be utilized to develop patient-specific perioperative plans with the goal of extending survival and limiting morbidity.

Beyond predictive analytics, developments in imaging technology have been utilized to improve intraoperative techniques. Recent work done by Yuan et al. studied hypertension-induced intracerebral hematoma external drainage techniques based on two new methods in a retrospective analysis: three-dimensional (3D) printing and laser guidance for navigation during puncture and debridement. In the 3D printing method, computed tomography (CT) image reconstructions were utilized to plan endoscope entry and trajectory into hematomas, which were then 3D printed in combination with patient face molds for customized intraoperative guidance. Laser guidance additionally utilized Xper-CT scans and 3D reconstructed images to emit a continuous focus directed towards the hematoma throughout the duration of the procedure. In direct intraoperative and postoperative comparisons, patients treated with 3D printing assistance were found to have significantly shorter operative durations than those treated with laser navigation assistance. However, outcomes including hematoma clearance rate, Glasgow Coma Sale improvement, and hospitalization time were similar between the two groups. The authors noted advantages to both techniques, including real-time navigation feedback and customized care, but may come at increased costs or extended preoperative setup time. Minimally invasive techniques in neurosurgery can be further improved with advancements in imaging and navigation, such as 3D printing and laser guidance.

Developments in image guidance technology have been additionally explored in cerebrospinal fluid (CSF) diversion approaches to reduce cerebellar contusion, such as in the study described by Roethlisberger et al. In their recent work, the authors utilized image-guided ipsilateral trigonal ventriculostomy for cerebellar pressure control during retrosigmoid craniotomy for cerebellopontine angle (CPA) tumor resection, after which outcomes were evaluated in a cohort of 52 patients. Prior to durotomy, the authors performed ventriculostomy of the ipsilateral trigone of the lateral ventricle using CT or magnetic resonance image (MRI) guidance, advancing an external ventricular drain (EVD) to release CSF and decompress the cerebellum. In the prospectively evaluated cohort, cerebellar swelling was avoided in 98% of cases using this technique. However, authors reported an 8% incidence of postoperative intracerebral or intraventricular hemorrhage without clinical complications. Ultimately, the authors recommended consideration of this technique for large posterior fossa tumors but not for small lesions.

New surgical methodologies for conditions previously lacking standardized treatment plans have been developed with the aid of technological advancements including endoscopic approaches. A novel transnasal endoscopic approach for treatment of traumatic oculomotor nerve palsy associated with superior orbital fissure fracture is described in a recent study by Wang et al. The authors of this work describe detailed anatomic landmarks encountered in their endoscopic approach, focusing on correct identification of the optic strut triangle, consisting of the internal carotid artery, optic nerve, and superior orbital fissure, in preparation for oculomotor nerve decompression. Hollowing of the optic strut by sequential drilling under endoscopic visualization achieved decompression of the optic and oculomotor nerves, resulting in rapid neurologic symptom improvement in 2 presented patient cases. Although still requiring further trials, the techniques described in this study may introduce alternatives to invasive approaches that arise with often extensive complications.

## Summary

Advancements in clinical and intraoperative technology have enabled neurosurgeons to create patient-specific management plans and improve postoperative outcomes for a range of conditions. In this research topic, four original studies were included that demonstrate the breadth of developments made in artificial intelligence-based predictive analytics, intraoperative imaging and navigation technologies, and novel cranial approaches aimed at improving patient outcomes. Further incorporation of machine learning algorithms, reconstructive software, and minimally invasive technology into daily medical practice will allow future generations of surgeons to provide personalized care to patients based on unique comorbidities and anatomic variations.

We certainly live in an era of rapid technologic advancement, but this also comes with great responsibility. As physicians and surgeons, it is our duty to question these technologies and understand their limitations before implementing them in our clinical practice. However, it is also our duty to embrace and use them when they can improve our patients’ lives. As our techniques and devices continue to evolve, we look forward to exploring the positive impacts made to neurosurgery and beyond.
